# Retraction Notice to "Congenital Double Scoliosis with 2 Bilaterally Alternant Supranumerary Hemivertebrae and 2 Supranumerary Vertebrae Treated by Growth Guiding Device" [J Med Life 12(2) (2019) 192-193]

**DOI:** 10.25122/jml-2020-1002

**Published:** 2020

**Authors:** Gheorghe Burnei, Viorel Tandea, Iuliu Liviu Muntean, Marius Moga, Ionut Daniel Raducan, Cristian Burnei

**Affiliations:** 1. "Carol Davila" University of Medicine and Pharmacy, Bucharest, Romania.; 2. Macta Clinic, Constanta, Romania.; 3. Tinos Clinic, Bucharest, Romania.; 4. Pediatric Surgery Department, "Grigore Alexandrescu" Clinical Emergency Hospital for Children, Bucharest, Romania.; 5. Pediatric Surgery and Orthopedy Department, Clinical Emergency Hospital for Children, Brasov, Romania.; 6. Orthopedy and Traumatology Department, "Dr. Carol Davila" Central Military Emergency University Hospital, Bucharest, Romania.; 7. "Vasile Goldis" Western University of Arad, Arad, Romania.; 8. Clinical Emergency Hospital, Bucharest, Romania.

## Abstract

Article J Med Life 2019; 12(2): 192-193; doi: 10.25122/jml-2019-0057 has been retracted for legal reasons.

## Retraction

The publisher has withdrawn this article [1] at the request of the parents/legal tutors of the patient under the General Data Protection Regulation (GDPR) of the European Union and Paragraph 26 of the Declaration of Helsinki version 7 2013.
